# Optimising the analysis of vascular prevention trials: Re-Assessment of the TARDIS trial, the first prevention trial to adopt an ordinal primary outcome measure^☆^

**DOI:** 10.1016/j.conctc.2023.101186

**Published:** 2023-07-05

**Authors:** Lisa J. Woodhouse, Alan A. Montgomery, Stuart Pocock, Marilyn James, Anna Ranta, Philip M. Bath, for the TARDIS Investigators

**Affiliations:** a Stroke Trials Unit, Mental Health & Clinical Neurosciences, School of Medicine, University of Nottingham, D Floor South Block, Queen's Medical Centre, Nottingham, NG7 2UH, UK; b Nottingham Clinical Trials Unit, University of Nottingham, Queen's Medical Centre, Derby Road, Nottingham, NG7 2UH, UK; c London School of Hygiene & Tropical Medicine, Keppel St., London, WC1E 7HT, UK; d Department of Medicine, University of Otago Wellington, Wellington, 6242, New Zealand; e Stroke, Nottingham University Hospitals NHS Trust, Queen's Medical Centre, Nottingham, NG7 2UH, UK

**Keywords:** TARDIS, Stroke, Severity, Outcome, Time

## Abstract

**Background:**

Ordinalised vascular outcomes incorporating event severity are more informative than binary outcomes that just include event numbers. The TARDIS trial was the first vascular prevention study to use an ordinalised vascular outcome as its primary efficacy and safety measures and collected severity information for other vascular events.

**Methods:**

TARDIS was an international prospective randomised open-label blinded-endpoint trial assessing one month of intensive versus guideline antiplatelet therapy in patients with acute non-cardioembolic stroke or TIA. Vascular events and their severity were recorded up to final follow-up at 90 days post randomisation. For each outcome, statistical techniques compared ordinal/continuous (10 models) and dichotomous (5 models) analyses; results were then ranked with the smallest p-value being given the smallest rank. Outcomes were also assessed within the pre-defined subgroup of participants with mild stroke (NIHSS≤3), or TIA recruited within 24 h.

**Results:**

Ordinal versions of vascular event outcomes were created in 3096 participants for stroke, myocardial infarction, major cardiac events, bleeding events, serious adverse events and venous thromboembolism (VTE), with 32 outcomes being created overall (29 in the subgroup population due to the absence of VTE events). Overall, the tests run on ordinal outcomes tended to rank higher than tests performed on binary outcomes. 764 (24.7%) participants were recruited within 24 h of a mild stroke/TIA; again, tests run on ordinal outcomes ranked higher.

**Conclusions:**

In TARDIS, tests performed on ordinal vascular outcomes tended to attain a higher rank than those performed on binary outcomes.

**Trial registration:**

ISRCTN47823388

## Highlights

• Vascular outcomes can be ordinal with the inclusion of severity information.
• Use of methods that analyse ordinal outcomes increases statistical efficiency.
• These approaches offer more information for patients and healthcare practitioners.
• Methods used here were relevant for majority of the tested vascular outcomes.

## 1 Background

The use of effective primary and secondary prevention strategies reduces the absolute risk of vascular events. As absolute event rates are a fundamental part of sample size calculations for binary events, a reduction in event numbers necessitates that clinical trials must be larger and/or run for longer time and so expose more patients to risks and be more expensive. [1] Further, there has also been an increase in the numbers of trials being undertaken as more therapies are developed and need testing. The combination of these two factors means that recruitment of the required number of patients is a challenging and competitive process. [2] New approaches therefore are needed to reduce the sample size of clinical trials, which in turn will reduce costs, completion times and the number of patients exposed to risks.

One approach would be to analyse vascular prevention trials in a way that does not lose clinically relevant data. For instance, vascular events, such as stroke or myocardial infarction, can be fatal or non-fatal. This means that 3-level ordinal outcomes (i.e., no event/non-fatal event/fatal event) could be analysed instead. Furthermore, depending on the event severity information collected, outcomes could potentially be extended to 4- or even 5-level outcomes. Analysis of this type of ordinal event outcome could be more efficient than that of binary outcomes, as it opens up the potential for reducing trial sample size, whilst also increasing the possibility of finding small clinically relevant benefits. This approach has been considered before for vascular prevention trials. [3] Additionally, the previous study conducted by the Optimising the Analysis of vascular Prevention trials (OA-Prevention) collaboration showed that ordinalised vascular event outcome, incorporating event severity (e.g. fatal/major/mild) should be considered and can be used as the primary measure, rather than the traditional binary event/no event outcome. [4]

The Triple Antiplatelets for Reducing Dependency after Ischaemic Stroke (TARDIS) trial was the first vascular prevention trial to prospectively use ordinal primary efficacy and safety outcome measures, and also recorded ordinal data for other outcomes. [5] The trial compared intensive antiplatelet therapy (combined aspirin, clopidogrel and dipyridamole) with guideline antiplatelet therapy (either combined aspirin and dipyridamole, or clopidogrel alone) in patients with acute non-cardioembolic ischaemic stroke (of any severity) or transient ischaemic attack (TIA) and recruited within 48 h of onset. Although intensive antiplatelet therapy did not reduce recurrence as compared with guideline therapy, a significant increase in bleeding was present with intensive antiplatelets. [5] Overall, the trial was neutral and there was no net balance in favour of hazard or benefit. These results differ from the Clopidogrel in High-Risk patients with Acute Non-disabling Cerebrovascular Events (CHANCE) trial and POINT trials which reported that treatment with combined aspirin and clopidogrel was associated with a lower risk of recurrent stroke events by 90 days in patients with minor ischaemic stroke or TIA when enrolled within 24 and 12 h of onset respectively. [6,7]

Here, we report a pre-planned re-analysis of the TARDIS trial comparing different ordinal and binary statistical approaches, using the same approaches as the prior OA-Prevention study. [4] We do this across the whole TARDIS population and then in the subgroup of participants that match those in CHANCE, i.e., those recruited within 24 h of a mild stroke (National Institutes of Health stroke scale, NIHSS≤3) or TIA. Statistical approaches used follow those previously reported in the prior OA-Prevention study. [4]

## 2 Methods

### 2.1 TARDIS design

TARDIS was a prospective, randomised, open-label, blinded-endpoint trial conducted across four countries at 106 sites. The protocol, statistical analysis plan, baseline characteristics of the participants and primary results have been published previously. [5,[8], [9], [10]] Briefly, adult patients aged 50 years or older were eligible for inclusion if they were at risk of a recurrent ischaemic event and had had either a non-cardioembolic ischaemic stroke (with limb weakness, dysphasia and/or hemianopia, and of any severity on the NIHSS) or a non-cardioembolic TIA (with at least 10 min of limb weakness or isolated dysphasia). Participants had to be randomised within 48 h of onset. TARDIS was approved by national and/or local ethics committees and national competent authorities in each participating country, was registered (ISRCTN47823388) and was adopted in the UK by the National Institute of Health Research (NIHR) Stroke Research Network. Participants gave written consent, or written proxy consent from a relative, carer or friend if they lacked capacity.

Participants were randomised to intensive antiplatelet therapy comprising combined aspirin (300 mg load then 50–150 mg daily), clopidogrel (300 mg load then 75 mg daily) and dipyridamole (200 mg twice daily modified release) or to guideline therapy comprising either clopidogrel alone or combined aspirin and dipyridamole; [5] the use of two control antiplatelet strategies reflected national guidelines and licenses at the time of the trial. Randomised antiplatelet drugs were then given for 30 days, after which participants were treated according to local guidelines.

### 2.2 Ordinal efficacy and safety outcomes

The final follow-up was performed centrally at 90 days by telephone from the coordinating centre in each country, with the assessor blinded to treatment allocation. The primary efficacy outcome comprised recurrence and its severity assessed using a six-level ordered categorical scale based in part on the modified Rankin Scale (mRS): fatal stroke (mRS 6), non-fatal major stroke (mRS 4 or 5), moderate stroke (mRS 2 or 3), mild stroke (mRS 0 or 1), TIA, and neither stroke nor TIA (Table 1). [5] Identification of recurrent cerebrovascular events was triangulated between reporting by the investigator, participant and general practitioner. The primary safety outcome was bleeding and its severity assessed using a five level ordered categorical scale: fatal, major, moderate, minor and none. [5] The definitions of fatal, major and moderate bleeding were according to the International Society on Thrombosis and Haemostasis and are based on severity, site of bleeding, fall in haemoglobin and need for transfusion. [11] Other outcomes collected or derived, with severity information, included myocardial infarction (MI), angina, cardiac events, venous thromboembolism (VTE), serious adverse events (SAEs) and major adverse cardiovascular events (MACE), as shown in Table 1. The primary efficacy and safety outcomes and SAEs (including cause-specific case-fatality) were validated and categorised by expert adjudicators who were blinded to treatment assignment. Participants who did not receive their allocated treatment or did not adhere to the protocol were still followed up in full at day 90 and included in all analyses.

**Table 1:** Ordered categorical severity levels for vascular event outcomes.

Outcome	Levels	Outcome format
Stroke, including TIA	4	Fatal/non-fatal/TIA/none
	5	Fatal/major/moderate-mild/TIA/none
(Primary outcome)	6	Fatal/major/moderate/mild/TIA/none
	9	Fatal/mRS = 5/mRS = 4/mRS = 3/mRS = 2/mRS = 1/mRS = 0/TIA/none
Stroke	3	Fatal/non-fatal/none
	4	Fatal/major/moderate-mild/none
	5	Fatal/major/moderate/mild/none
	8	Fatal/mRS = 5/mRS = 4/mRS = 3/mRS = 2/mRS = 1/mRS = 0/none
MI	3	Fatal/non-fatal/none
	4	Fatal/major/moderate-mild/none
	5	Fatal/major/moderate/minor/none
MI, including angina	4^a^	Fatal/non-fatal/angina/none
	5^a^	Fatal/major/moderate-mild/angina/none
	5^b^	Fatal/non-fatal/unstable angina/stable angina/none
	6^a^	Fatal/major/moderate/mild/angina/none
	6^b^	Fatal/major/moderate-mild/unstable angina/stable angina/none
	7^b^	Fatal/major/moderate/mild/unstable angina/stable angina/none
Bleeding event	3	Fatal/non-fatal/none
	4	Fatal/major/moderate-mild/none
	5	Fatal/major/moderate/minor/none
Cardiac event	3	Fatal/non-fatal/none
	4	Fatal/major/moderate-mild/none
	5	Fatal/major/moderate/minor/none
SAE	3	Fatal/non-fatal/none
	4	Fatal/major/moderate-mild/none
	5	Fatal/major/moderate/minor/none
MACE	3	Fatal/non-fatal/none
	4	Fatal/major/moderate-mild/none
	5	Fatal/major/moderate/minor/none

MI: Myocardial infarction; MACE: Major cardiovascular events; mRS: modified Rankin Scale; SAE: Serious adverse event; TIA: Transient ischaemic attack; VTE: Venous thromboembolism.

a Includes a composite of stable and unstable angina as a level.

b Includes stable and unstable angina as separate levels.

### 2.3 Mild stroke/TIA

A pre-specified sub-group of participants matching those in CHANCE was created as those with an index event of mild stroke (NIHSS<4) or TIA with randomisation within 24 h. [6]

### 2.4 Novel ordinal outcomes

In addition to the 6-level primary efficacy and 5-level primary safety outcomes, [5] we created further ordered categorical outcomes including 3 to 6, 8 and 9-level stroke/TIA measures, 3 to 7 level myocardial infarction/angina events, and 3 to 5 level events relating to bleeding, major adverse cardiac events (MACE), venous thromboembolism (VTE) and serious adverse events (SAEs) (Table 1). These ranged in each case between no event and a fatal outcome.

### 2.5 Statistical testing

Treatment effect (intensive versus guideline) on outcome was assessed using methods for binary and/or ordered categorical outcomes. Methods used included the Chi-Square test (used on binary outcomes, binary fatal outcomes and ordinal outcomes), binary logistic regression (BLR, adjusted, used on binary outcomes), Cox proportional hazards models (CPH, adjusted and unadjusted, used on binary outcomes), Cochran-Armitage Trend test (used on ordinal outcomes), Mann-Whitney *U* test (used on ordinal outcomes), median test (used on ordinal outcomes), ordinal logistic regression (OLR, adjusted and unadjusted, used on ordinal outcomes), bootstrapping the mean rank (used on ordinal outcomes), *t*-test (used on ordinal outcomes), multiple linear regression (MLR, adjusted, used on ordinal outcomes) and the Win Ratio test. [[12], [13], [14], [15], [16]] The Win ratio test is a method where multiple outcomes, with varying levels of clinical importance (e.g. fatal stroke, non-fatal stroke, TIA), can be analysed together to determine a ‘Win ratio’ (calculated as wins/losses). For this approach we created and analysed multiple binary outcomes based on our ordered categorical outcomes (see Table 1), and the severity of the event was used to determine the clinical importance. These methods were chosen as they were implemented in the prior OA-Prevention study publication. [4] Of these methods, two, Cox Proportional Hazards models and the Win Ratio test, utilized the timing of the event. Typically, the *t*-test and MLR are not used on ordinal outcome data but were used in this instance due to researcher familiarity and for comparison with more traditional ordinal analysis methods. An overview of these methods can be seen in Supplementary Table 1. For the adjusted analyses the covariates comprised age, sex, pre-morbid function, systolic blood pressure, syndrome (OSCP classification), previous antiplatelet therapy (number of tablets), use of gastroprotection, use of low dose heparin, time to randomisation, NIHSS and treatment with alteplase. [5]

### 2.6 Comparisons of analysis methods

For each ordinal event outcome constructed (Table 1), binary cuts (e.g., Any event/No event, Fatal event/No Fatal event, Non-fatal event/No Non-fatal event) of the outcome were also created for use in the Win ratio test and the binary analysis methods. Treatment effect on outcome was then compared using each of the ordinal/continuous outcome analysis methods on the ordinal event outcome and using the appropriate binary analysis methods on the counterpart binary cuts. The resulting p-values of these methods were then ranked, in ascending order, with the method with the smallest p-value being given the highest rank of 1 and so on. An example of how this analysis was undertaken can be seen in Supplementary Table 2. Once the ranking of the tests had been determined for each outcome comparison the results were then tabulated and displayed as a heat map of rankings. This made it possible to determine any trends in the ranking of tests. The proportion of significant results (p < 0.05) for each test, across the different outcomes, was also calculated.

### 2.7 Statistical analyses

Data are number (%), median [25th, 75th quartiles] or mean (standard deviation, SD). No adjustment was made for multiplicity of testing for secondary analyses. All analyses were by the intention to treat principle for all comparisons. No imputation was performed in the case of missing data. Statistical analyses were performed using SAS software (versions 9.4).

## 3 Results

### 3.1 Study participants

TARDIS commenced recruitment on 7^th^ April 2009 but was halted on the 18^th^ March 2016 on the advice of the trial's independent Data Monitoring Committee (DMC). [5] The DMC reported a significant increase in major bleeding without a significant reduction in stroke. In the published analysis, the absolute numbers of stroke and bleeding events were similar. The rate of TIA was lower with triple antiplatelet therapy. Of the 3096 participants (intensive group 1556 [50.3%]; guideline 1540 [49.7%]), 2143 participants (69.2%) were recruited with a diagnosis of ischaemic stroke with the remaining 30.8% having a TIA (Table 2). [5] The median time from onset to recruitment was 29.3 h [quartiles: 21.8, 39.6 h] with 764 (24.7%) recruited within 24 h of a mild stroke (305, 39.9%) or TIA (459, 60.1%).

**Table 2:** Baseline characteristics.

	TARDIS			Mild/TIA ≤24-h subgroup		
	All	Intensive	Guideline	All	Intensive	Guideline
	3096	1556	1540	764	385	379
Demographics						
Age (years)	69.0 (10.1)	69.1 (9.9)	68.9 (10.3)	69.0 (10.0)	69.3 (9.7)	68.8 (10.2)
Sex, male (%)	1945 (63)	982 (63)	963 (63)	482 (63)	256 (66)	226 (60)
mRS, pre-morbid	0.0 [0.0, 0.0]	0.0 [0.0, 0.0]	0.0 [0.0, 0.0]	0.0 [0.0, 0.0]	0.0 [0.0, 0.0]	0.0 [0.0, 0.0]
Time OTR (hours)	29.3 [21.8, 39.6]	29.3 [21.7, 39.7]	29.3 [21.9, 39.5]	17.3 [9.5, 21.5]	17.7 [10.5, 21.6]	16.9 [8.9, 21.5]
Medical history (%)						
Smoking, current	784 (26)	404 (26)	380 (25)	151 (20)	72 (19)	79 (21)
Alcohol, heavy	291 (10)	150 (10)	141 (9)	66 (9)	34 (9)	32 (9)
Hypertension	1824 (59)	930 (60)	894 (58)	440 (58)	225 (58)	215 (57)
Hyperlipidaemia	1317 (44)	655 (44)	662 (45)	344 (47)	161 (43)	183 (51)
Diabetes	590 (19)	280 (18)	310 (20)	142 (19)	65 (17)	77 (20)
Atrial fibrillation	1 (0)	0 (0)	1 (0)	0 (0)	0 (0)	0 (0)
Stroke	348 (11)	189 (12)	159 (10)	75 (10)	46 (12)	29 (8)
Ischaemic heart disease	403 (13)	196 (13)	207 (13)	83 (11)	39 (10)	44 (12)
Peripheral artery disease	70 (2)	40 (3)	30 (2)	16 (2)	7 (2)	9 (2)
Bleed, major	19 (1)	9 (1)	10 (1)	4 (1)	2 (1)	2 (1)
Within 12 months	0 (0)	0 (0)	0 (0)	0 (0)	0 (0)	0 (0)
Clinical features (%)						
NIHSS (/42)	2.8 (3.6)	2.9 (3.7)	2.7 (3.5)	1.0 (1.2)	1.0 (1.3)	0.9 (1.2)
OCSP, TACI (%)	181 (6)	86 (6)	95 (6)	6 (1)	4 (1)	2 (1)
Systolic BP (mmHg)	143.5 (18.2)	143.4 (17.8)	143.6 (18.5)	145.9 (18.0)	146.2 (17.7)	145.5 (18.3)
Weight (kg approx.)	75.3 (16.6)	75.5 (16.7)	75.1 (16.5)	75.8 (16.4)	76.2 (16.2)	75.4 (16.6)
Diagnostic (%)						
Qualifying event (%)						
Ischaemic stroke	2143 (69)	1076 (69)	1067 (69)	305 (40)	152 (39)	153 (40)
TIA	953 (31)	480 (31)	473 (31)	459 (60)	233 (61)	226 (60)
Treatment (%)						
Prior AP(s)	1080 (35)	557 (36)	523 (34)	262 (34)	137 (36)	125 (33)
Prior heparin	7 (0)	2 (0)	5 (0)	1 (0)	0 (0)	1 (0)
Alteplase	341 (11)	169 (11)	172 (11)	1 (0)	0 (0)	1 (0)
AP + Alteplase	196 (6)	97 (6)	99 (6)	0 (0)	0 (0)	0 (0)
Randomised AP						
ACD	1556 (50)	1556 (100)	0 (0)	385 (50)	385 (100)	0 (0)
AD	691 (22)	0 (0)	691 (45)	204 (27)	0 (0)	204 (54)
Clopidogrel	849 (27)	0 (0)	849 (55)	175 (23)	0 (0)	175 (46)
Imaging (%)						
Compatible lesion	1051 (40)	533 (40)	518 (39)	156 (26)	70 (24)	86 (28)
Mass effect	514 (49)	266 (50)	248 (48)	70 (45)	35 (50)	35 (41)
ASPECTS [/10]	8.0 [7.0, 9.0]	8.0 [7.0, 9.0]	8.0 [7.0, 9.0]	9.0 [7.0, 9.0]	9.0 [7.0, 9.0]	8.0 [7.0, 9.0]
Atrophy	2448 (93)	1218 (92)	1230 (93)	576 (95)	280 (95)	296 (95)
Leukoaraiosis	1054 (40)	534 (41)	520 (40)	221 (36)	110 (37)	111 (36)
Old stroke	1595 (61)	807 (61)	788 (60)	349 (57)	173 (58)	176 (56)
Frailty score [/3]	2.0 [1.0, 3.0]	2.0 [1.0, 3.0]	2.0 [1.0, 3.0]	2.0 [1.0, 2.0]	2.0 [1.0, 2.0]	2.0 [1.0, 2.0]

ACD: Aspirin, Clopidogrel & Dipyridamole; AD: Aspirin & Dipyridamole; AP: antiplatelets; ASPECTS: Alberta stroke programme early CT score; OTR: onset to randomisation; TIA: transient ischaemic attack.

### 3.2 Outcomes and ranks of tests – All participants

Overall, 3070 (99.2%) participants had data relating to stroke, TIA, MI, angina, cardiac events, MACE and VTE, 3072 (99.2%) for bleeding and 3074 (99.3%) for SAEs (Table 3).

**Table 3:**
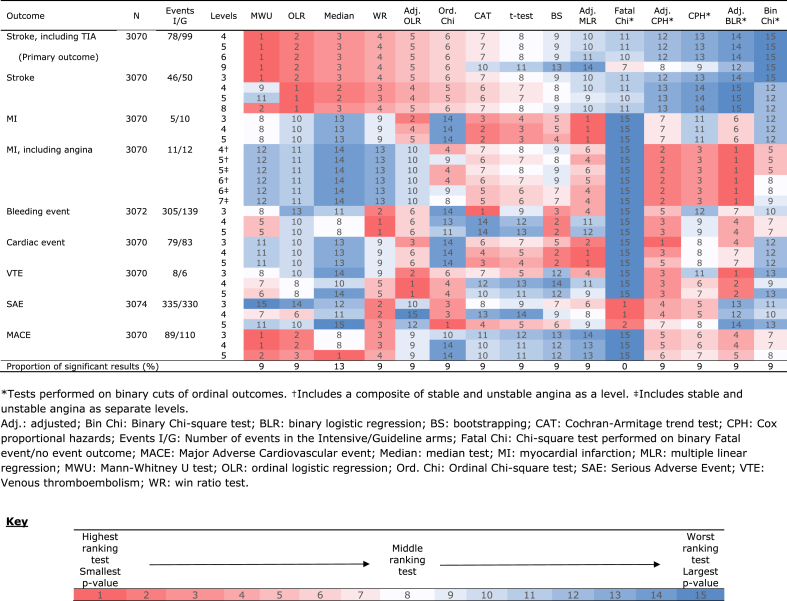
Heat map showing rankings of tests for each outcome in all participants.

The results of the rankings of the tests for each of the 32 outcome comparisons can be seen in Table 3. For events such as stroke including TIA (all levels), stroke (3-level) and MACE (3- and 4-level), the Mann-Whitney *U* test had the smallest rank. Adjusted MLR was the test with the smallest rank for events including MI (all levels – not including angina) and cardiac events (4- and 5-level). OLR, was the test with the highest rank for comparisons undertaken on the 4-, 5- and 8-level stroke outcomes. Adjusted OLR was the test with the smallest rank for the 4- and 5-level VTE outcomes. The Median test, ordinal Chi-Square test and Cochran-Armitage trend test each had the highest rank for events including MACE (5-level), SAEs (5-level) and bleeding (3-level) respectively. Adjusted BLR was the test with the smallest rank for all MI including angina outcome comparisons and the 3-level VTE comparison.

The test with the largest proportion of significant results was the Median test with 13% (Table 3). The rest of the tests, with the exception of the fatal Chi-Square, had a 9% significant proportion rate. The number of significant results for each test can be seen in Supplementary Table 3.

### 3.3 Outcomes and ranks of tests – the minor stroke/TIA, ≤24-h subgroup

Out of the 764 participants in this subgroup, 755 (98.8%) had outcome data regarding stroke, TIA, MI, angina, cardiac events and MACE (Table 4). 756 (99.0%) participants had data regarding bleeding events and SAEs. It was not possible to perform outcome analyses for VTE as no events occurred in this population of participants.

**Table 4:**
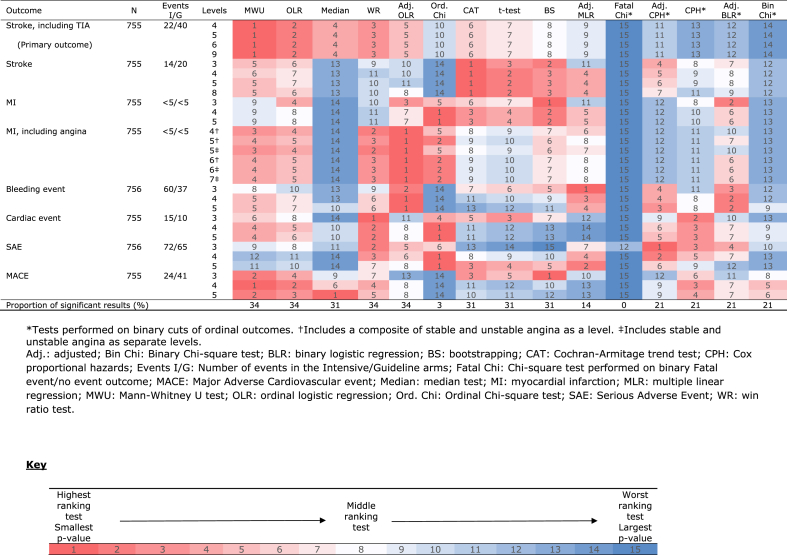
Heat map showing rankings of tests for each outcome in Minor stroke/TIA participants recruited within 24 h.

The results of the rankings of the tests for each of the 32 outcome comparisons can be seen in Table 4. Adjusted OLR was the test with the smallest rank, for outcomes such as MI including angina (all levels) and bleeding events (4- and 5-level). The ordinal Chi-Square test was the test with the highest rank for event outcomes including MI (4- and 5-level), cardiac events (4- and 5-level) and SAEs (4- and 5-level). As for The Mann-Whitney *U* test, this test had the highest rank for event outcomes such as stroke including TIA (all levels) and MACE (4-level). The test with the highest rank for all 5 stroke outcomes was the Cochran-Armitage trend test. Bootstrapping was then the test with the highest rank for outcome comparisons relating to 3-level MI and 3-level MACE. As for MLR and the Median test, these methods were each the test with the highest rank for the comparisons including the 3-level bleeding and 5-level MACE outcomes respectively.

The tests with the largest proportions of significant results were Mann-Whitney *U* test, adjusted and unadjusted ordinal logistic regression and the Win Ratio test, each having a proportion of 34% (Table 4). The number of significant results for each test can be seen in Supplementary Table 4.

## 4 Discussion

We created 32 ordinal versions of vascular event outcomes for stroke, MI, bleeding events, cardiac events, MACE, SAEs and venous thromboembolism. For both TARDIS overall and in participants with mild stroke/TIA, the tests performed on the ordinal vascular outcomes tended to tended to rank higher than binary outcome analyses; the Mann-Whitney *U* test, ordinal logistic regression, Win Ratio, Cochran-Armitage trend test and adjusted multiple linear regression all were associated with the smallest ranks for at least some outcomes. Comparing the statistical tests in the subgroup of participants with minor stroke or TIA and randomised within 24 h was important since these parameters matched the positive CHANCE trial. [6] We did not assess the population adhering to the POINT trial inclusion criteria [7] since few TARDIS participants were recruited within 12 h.

The idea that the use of a binary/dichotomised scale as an outcome measure is not always suitable has been considered before in other areas of research. Areas where this has been tested before, for other event outcomes and also outcome scales, are quality of hospital care, [17] compensated cirrhosis, [18] severe influenza, [19] quality of life outcome measures, [20,21] traumatic brain injury trials [22] and acute stroke trials. [23,24] These studies also found that ordinal analyses tended to rank higher than binary methods, and in some instances time-to-event methods. Ordinal analysis methods appeared to provide increased statistical power to detect differences, thus requiring fewer patients for the final analysis. This would suggest that use of an ordinal outcome measure as the primary outcome should be considered as a possible approach by trialists if applicable. Use of this type of outcome could reduce the required sample size for a trials and could also be more informative to participants and healthcare professionals than binary ones. [3] For example, in the case of event outcomes, rather than saying that an intervention reduces the risk of an event, we could say that it reduces both occurrence and also the severity of the event.

From an implementation perspective, trialists should consider the prospective collection of information on event severity as well as event numbers for stroke, MI, MACE/cardiac events, VTE, bleeding and adverse events; theoretically, these approaches should work for other medical conditions that have ordered categorical scales such as heart, liver, renal and respiratory failure. In general, this will enhance the statistical power of analyses (or allow smaller sample sizes). Unadjusted analyses can use the MWU or Win Ratio tests; similarly, covariate analyses can use OLR. This study would recommend the use of adjusted OLR (or generalised OLR if the proportionality of odds assumption fails) for the analysis of ordered categorical event outcomes. This is due to the fact that parametric tests are more powerful than their non-parametric equivalents, it is possible to adjust for important prognostic covariates and this test it capable of producing a meaningful estimate of effect (i.e., odds ratio and confidence intervals) that researchers will be familiar with. To date, TARDIS remains the only vascular prevention trial to have prospectively chosen an ordinal outcome although secondary or post hoc analyses of ordered categorical outcomes have been reported previously. [3,[25], [26], [27], [28], [29]]

This analysis had several strengths, for instance, TARDIS had a large sample size (>3000 participants), concealment of treatment assignment and assessment prospectively of multiple event outcomes. The trial also collected data regarding patient characteristics thereby allowing for covariate adjusted analyses. Lastly, ordinal outcomes have embedded dichotomies for worse versus better outcome. For instance, event versus no event, major event versus no major event and so on. This means that if statistical significance is shown, further closed testing methods can be applied to present results for the important dichotomies.

However, there were limitations. Firstly, treatment was administered in an open-label design which could have increased the reporting of known adverse events. Secondly, recruitment to the TARDIS trial was stopped early following recommendation by the independent data monitoring committee which will have impacted the statistical power. Third, some of statistical methods used for the analysis of the ordinal outcomes are not completely relevant as they are more commonly used for continuous outcome analysis (*t*-test, multiple linear regression). Fourth, the statistical methods used for this analysis vary in their design, which could affect the comparison of analyses. Nevertheless, these methods were included as they are familiar to researchers and are readily available in statistical textbooks and analysis software. Fifth, the rankings of tests appear to be correlated within each outcome category (e.g., stroke, MI, MACE). Therefore, it could be suggested that the results presented may be dominated by rankings from particular outcome categories. Last, another limitation is the use of p-values to compare the methods and draw conclusions, as evidence-based decision-making is not predominantly influenced by p-values. However, this approach was necessary since different statistical methods produce different quantifying effects and p-values are a common output. Additionally, the ranking of p-values within comparisons is very similar to the ranking of standardised effect sizes.

## 5 Conclusions

In summary, this analysis shows that vascular outcomes can be ordinal with the inclusion of severity information. This approach, if used, could be beneficial as it will provide further information for participants, public and healthcare practitioners. The methods used appeared to be relevant for the majority of the tested vascular outcomes.

## Author contributions

LJW, PMB and AAM are responsible for the design of the study. LJW conducted the analyses and drafted the manuscript. All authors (LJW, AAM, SP, MJ, AR and PMB) commented on the analyses and drafts of this report and have seen and approved the final version.

## Funding

The TARDIS start-up phase was funded by the 10.13039/501100000274British Heart Foundation (grant PG/08/083/25779, 1 April 2009 – 30 September 2012). The 10.13039/501100000272NIHR 10.13039/501100000664HTA programme funded the main phase of this project (10/104/24, 1 October 2012 – 30 September 2017, http://www.nets.nihr.ac.uk/projects/hta/1010424). The views expressed are those of the author(s) and not necessarily those of the NHS, the NIHR or the Department of Health. Indirect funding was provided by The 10.13039/501100000364Stroke Association through its funding of the Stroke Trials Unit, 10.13039/501100000837University of Nottingham. The trial was sponsored by the 10.13039/501100000837University of Nottingham.

## Data sharing

Individual participant data from the TARDIS trial will be shared with the Virtual International Stroke Trials Archive (VISTA) collaboration. The Chief Investigator (with approval from the Trial Steering Committee as necessary) will consider other requests to share individual participant data via email at: tardis@nottingham.ac.uk. Requests will require a protocol detailing the hypothesis, aims, analyses, and intended tables and figures. Where possible, we will perform the analyses; alternatively, de-identified data and a data dictionary will be supplied for the necessary variables for remote analysis. Any sharing will be subject to a signed data access agreement. Ultimately, the data will be published.

## Declaration of competing interest

The authors declare the following financial interests/personal relationships which may be considered as potential competing interests: PMB is Stroke Association Professor of Stroke Medicine and is a NIHR Senior Investigator; he was Chief Investigator for the TARDIS trial, and has consulted with DiaMedica, Moleac and Phagenesis. LJW was funded in part by NIHR TARDIS (10/104/24). AAM, SP, MJ and AR report no conflicts of interest.

## Data Availability

The data are shared with the VISTA Collaboration
