# Supplementary material for: Optimising the analysis of vascular prevention trials: Re-Assessment of the TARDIS trial, the first prevention trial to adopt an ordinal primary outcome measure
Source: Contemp Clin Trials Commun. 2023 Jul 5;35:101186. doi: 10.1016/j.conctc.2023.101186 (PMC10517366; doi:10.1016/j.conctc.2023.101186)
Supplement: Multimedia component 1 [file mmc1.docx]

**Optimising the analysis of vascular prevention trials: re-assessment of the TARDIS trial, the first prevention trial to adopt an ordinal primary outcome measure.**

**SUPPLEMENTARY APPENDIX**

**Writing Committee**

Lisa J Woodhouse;^1^ Alan A Montgomery;^2^ Stuart Pocock;^3^ Marilyn James;^2^ Anna Ranta;^4^ and Philip M Bath;^1, 5^ for the TARDIS Investigators

1. Stroke Trials Unit, Mental Health & Clinical Neurosciences, University of Nottingham, D Floor South Block, Queen’s Medical Centre, Nottingham NG7 2UH, UK
2. Nottingham Clinical Trials Unit, University of Nottingham, Queen’s Medical Centre, Derby Road, Nottingham NG7 2UH, UK
3. London School of Hygiene & Tropical Medicine, Keppel St., London WC1E 7HT, UK
4. Department of Medicine, University of Otago Wellington, Wellington 6242, New Zealand
5. Stroke, Nottingham University Hospitals NHS Trust, Queen’s Medical Centre, Nottingham NG7 2UH, UK

Trial registration: ISRCTN47823388

TARDIS was funded by National Institutes of Health Research Health Technology Assessment Programme and British Heart Foundation

Table 1. Overview of statistical analysis methods

| **Analysis method** | **Outcome type** | **Statistical assumptions** | **Advantages** | **Disadvantages** |
| --- | --- | --- | --- | --- |
| Binary logistic regression  (BLR) | Binary | - No assumptions made about explanatory variables | - Can adjust for covariates | - Large number of observations required |
| Cox proportional hazards  (CPH) | Binary | - Proportionality of hazards over time - Censoring of observations is unrelated to prognosis | - Can adjust for covariates | - If assumptions of the model not met then subsequent analyses and risk estimates will possibly be biased |
| Chi-square (χ^2^)  (CS) | Binary and ordered categorical | - Chi-Square – Total count is >40 or total count is 20-40 and the expected value of each exposure-outcome category is >5 | - Simple to implement | - Cannot adjust for covariates |
| Cochran-Armitage trend test (CAT) | Ordered categorical | - Similar to the Chi-square test but it takes into account the ordering across categories | - Easy to interpret | - Cannot adjust for covariates |
| Ordinal logistic regression  (OLR) | Ordered categorical | - Response is ordinal - Proportionality of odds | - Can adjust for covariates | - If assumptions of the model not met then subsequent analyses and odds estimates will possibly be biased |
| Mann-Whitney U test  (MWU) | Ordered categorical | - Non-parametric test - Response is ordinal / continuous - Observations from both groups are independent of one another | - Easy to interpret | - Cannot adjust for covariates – there are extensions of this method, which allow for adjustment (34-36) |
| Median test  (MT) | Ordered categorical | - Non-parametric test - Considers the position of each observation relative to the overall median. | - Easy to interpret | - Cannot adjust for covariates - Inefficient (low power) to detect differences if sample size is large. |
| t-test | Continuous (used on the ordered categorical) | - Homogeneity of variances | - Easy to interpret | - Cannot adjust for covariates |
| Multiple linear regression  (MLR) | Continuous (used on the ordered categorical) | - Linear relationship - Homogeneity of variances - No or little multicollinearity | - Can adjust for covariates | - Assumes linear relationship - Sensitive to outliers |
| Win Ratio test  Wins/losses version  (WR) | Combination of multiple outcomes | - Accounts for clinical priorities of endpoints | - Prioritises the most severe outcome - Useful for composite outcomes - Extensions of this approach allow for covariate adjustment - Easy to interpret | - Doesn’t use the precise times from randomisation to event occurrence |
| Bootstrapping  (BS) | Ordered categorical | - None | - No assumptions made about the distribution of the data | - Cannot adjust for covariates - Computationally intensive - Doesn’t provide a meaningful point estimate |

Table 2. Example analysis process – based on 4-level Event outcome analysis process (fatal event/ severe event/ minor event/ no event).

| 1. For each of the participants with available data regarding the event and severity (e.g., fatal, non-fatal severe, non-fatal minor, no event) the following outcomes were created:    1. **Ordinal 4-level**– analysed using the following approaches: Unadjusted – ordinal logistic regression, Mann-Whitney U test, Cochran-Armitage trend test, Median test, Chi-Square test, t-test, and bootstrapping of the mean rank; Adjusted – ordinal logistic regression and multiple linear regression.    2. **Binary (any event/no event)** – analysed using the following approaches: Unadjusted – Chi-Square test, Cox Proportional hazards (incorporating time to first event); Adjusted – binary logistic regression and Cox proportional hazards (incorporating time to first event)    3. **Binary (fatal event/ no fatal event)** – analysed using Chi-Square test and used for the Win ratio test    4. **Binary (severe event/ no severe event)** – for use in the Win ratio test only    5. **Binary (minor event/ no minor event)** – for use in Win ratio test only | | | | | | | | | | | | | | | |
| --- | --- | --- | --- | --- | --- | --- | --- | --- | --- | --- | --- | --- | --- | --- | --- |
| 1. All of the tests are performed on the corresponding outcome(s) and the p-values are extracted to a new dataset, e.g. | | | | | | | | | | | | | | | |
| **Analysis** | **Chi-Square (Fatal)** | **Chi-Square (Binary)** | **Adjusted BLR** | **Adjusted CPH** | **Adjusted OLR** | **Adjusted MLR** | **Un-adjusted CPH** | **C-A trend test** | **Chi-Square (Ordinal)** | **Un-adjusted OLR** | **t-test** | **Mann-Whitney U** | **Median test** | **Boot-strapping** | **Win ratio** |
| Event 4l | 0.91 | 0.0074 | 0.0052 | 0.0039 | 0.0011 | 0.0027 | 0.0049 | 0.0031 | 0.43 | 0.0014 | 0.0030 | 0.0013 | 0.0017 | 0.0037 | 0.024 |
| 1. Then each of the p-values is ranked 1 to 15, from smallest to largest, e.g. | | | | | | | | | | | | | | | |
| **Analysis** | **Chi-Square (Fatal)** | **Chi-Square (Binary)** | **Adjusted BLR** | **Adjusted CPH** | **Adjusted OLR** | **Adjusted MLR** | **Un-adjusted CPH** | **C-A trend test** | **Chi-Square (Ordinal)** | **Un-adjusted OLR** | **t-test** | **Mann-Whitney U** | **Median test** | **Boot-strapping** | **Win ratio** |
| Event 4l | 15 | 12 | 11 | 9 | 1 | 5 | 10 | 7 | 14 | 3 | 6 | 2 | 4 | 8 | 13 |
| 1. This process, with adjustment depending on number of levels, is performed for each ordinal outcome in Table 1. | | | | | | | | | | | | | | | |
| **Analysis** | **Chi-Square (Fatal)** | **Chi-Square (Binary)** | **Adjusted BLR** | **Adjusted CPH** | **Adjusted OLR** | **Adjusted MLR** | **Un-adjusted CPH** | **C-A trend test** | **Chi-Square (Ordinal)** | **Un-adjusted OLR** | **t-test** | **Mann-Whitney U** | **Median test** | **Boot-strapping** | **Win ratio** |
| Event 4l | 15 | 12 | 11 | 9 | 1 | 5 | 10 | 7 | 14 | 3 | 6 | 2 | 4 | 8 | 13 |
| Event Xl | 15 | 13 | 10 | 11 | 3 | 6 | 12 | 5 | 14 | 2 | 7 | 1 | 8 | 9 | 4 |
| $\vdots$ | $\vdots$ | $\vdots$ | $\vdots$ | $\vdots$ | $\vdots$ | $\vdots$ | $\vdots$ | $\vdots$ | $\vdots$ | $\vdots$ | $\vdots$ | $\vdots$ | $\vdots$ | $\vdots$ | $\vdots$ |

Table 3. P-values of tests for each outcome in all participants.

| Outcome | N | Levels | MWU | OLR | Median | WR | Adj. OLR | Ord. Chi | CAT | t-test | BS | Adj. MLR | Fatal Chi* | Adj. CPH* | CPH* | Adj. BLR* | Bin Chi* |
| --- | --- | --- | --- | --- | --- | --- | --- | --- | --- | --- | --- | --- | --- | --- | --- | --- | --- |
| Stroke, including TIA | 3070 | 4 | ~ | ~ | ~ | ~ | ~ | ~ | ~ | ~ | ~ | ~ | ~ | ~ | ~ | ~ | ~ |
|  |  | 5 | ~ | ~ | ~ | ~ | ~ | ~ | ~ | ~ | ~ | ~ | ~ | ~ | ~ | ~ | ~ |
| (Primary outcome) |  | 6 | ~ | ~ | ~ | ~ | ~ | ~ | ~ | ~ | ~ | ~ | ~ | ~ | ~ | ~ | ~ |
|  |  | 9 | ~ | ~ | ~ | ~ | ~ | ~ | ~ | ~ | ~ | ~ | ~ | ~ | ~ | ~ | ~ |
| Stroke | 3070 | 3 | ~ | ~ | ~ | ~ | ~ | ~ | ~ | ~ | ~ | ~ | ~ | ~ | ~ | ~ | ~ |
|  |  | 4 | ~ | ~ | ~ | ~ | ~ | ~ | ~ | ~ | ~ | ~ | ~ | ~ | ~ | ~ | ~ |
|  |  | 5 | ~ | ~ | ~ | ~ | ~ | ~ | ~ | ~ | ~ | ~ | ~ | ~ | ~ | ~ | ~ |
|  |  | 8 | ~ | ~ | ~ | ~ | ~ | ~ | ~ | ~ | ~ | ~ | ~ | ~ | ~ | ~ | ~ |
| MI | 3070 | 3 | ~ | ~ | ~ | ~ | ~ | ~ | ~ | ~ | ~ | ~ | ~ | ~ | ~ | ~ | ~ |
|  |  | 4 | ~ | ~ | ~ | ~ | ~ | ~ | ~ | ~ | ~ | ~ | ~ | ~ | ~ | ~ | ~ |
|  |  | 5 | ~ | ~ | ~ | ~ | ~ | ~ | ~ | ~ | ~ | ~ | ~ | ~ | ~ | ~ | ~ |
| MI, including angina | 3070 | 4† | ~ | ~ | ~ | ~ | ~ | ~ | ~ | ~ | ~ | ~ | ~ | ~ | ~ | ~ | ~ |
|  |  | 5† | ~ | ~ | ~ | ~ | ~ | ~ | ~ | ~ | ~ | ~ | ~ | ~ | ~ | ~ | ~ |
|  |  | 5‡ | ~ | ~ | ~ | ~ | ~ | ~ | ~ | ~ | ~ | ~ | ~ | ~ | ~ | ~ | ~ |
|  |  | 6† | ~ | ~ | ~ | ~ | ~ | ~ | ~ | ~ | ~ | ~ | ~ | ~ | ~ | ~ | ~ |
|  |  | 6‡ | ~ | ~ | ~ | ~ | ~ | ~ | ~ | ~ | ~ | ~ | ~ | ~ | ~ | ~ | ~ |
|  |  | 7‡ | ~ | ~ | ~ | ~ | ~ | ~ | ~ | ~ | ~ | ~ | ~ | ~ | ~ | ~ | ~ |
| Bleeding event | 3072 | 3 | ++++ | ++++ | ++++ | ++++ | ++++ | ++++ | ++++ | ++++ | ++++ | ++++ | ~ | ++++ | ++++ | ++++ | ++++ |
|  |  | 4 | ++++ | ++++ | ++++ | ++++ | ++++ | ++++ | ++++ | ++++ | ++++ | ++++ | ~ | ++++ | ++++ | ++++ | ++++ |
|  |  | 5 | ++++ | ++++ | ++++ | ++++ | ++++ | ++++ | ++++ | ++++ | ++++ | ++++ | ~ | ++++ | ++++ | ++++ | ++++ |
| Cardiac event | 3070 | 3 | ~ | ~ | ~ | ~ | ~ | ~ | ~ | ~ | ~ | ~ | ~ | ~ | ~ | ~ | ~ |
|  |  | 4 | ~ | ~ | ~ | ~ | ~ | ~ | ~ | ~ | ~ | ~ | ~ | ~ | ~ | ~ | ~ |
|  |  | 5 | ~ | ~ | ~ | ~ | ~ | ~ | ~ | ~ | ~ | ~ | ~ | ~ | ~ | ~ | ~ |
| VTE | 3070 | 3 | ~ | ~ | ~ | ~ | ~ | ~ | ~ | ~ | ~ | ~ | ~ | ~ | ~ | ~ | ~ |
|  |  | 4 | ~ | ~ | ~ | ~ | ~ | ~ | ~ | ~ | ~ | ~ | ~ | ~ | ~ | ~ | ~ |
|  |  | 5 | ~ | ~ | ~ | ~ | ~ | ~ | ~ | ~ | ~ | ~ | ~ | ~ | ~ | ~ | ~ |
| SAE | 3074 | 3 | ~ | ~ | ~ | ~ | ~ | ~ | ~ | ~ | ~ | ~ | ~ | ~ | ~ | ~ | ~ |
|  |  | 4 | ~ | ~ | ~ | ~ | ~ | ~ | ~ | ~ | ~ | ~ | ~ | ~ | ~ | ~ | ~ |
|  |  | 5 | ~ | ~ | ~ | ~ | ~ | ~ | ~ | ~ | ~ | ~ | ~ | ~ | ~ | ~ | ~ |
| MACE | 3070 | 3 | ~ | ~ | ~ | ~ | ~ | ~ | ~ | ~ | ~ | ~ | ~ | ~ | ~ | ~ | ~ |
|  |  | 4 | ~ | ~ | ~ | ~ | ~ | ~ | ~ | ~ | ~ | ~ | ~ | ~ | ~ | ~ | ~ |
|  |  | 5 | ~ | ~ | ++++ | ~ | ~ | ~ | ~ | ~ | ~ | ~ | ~ | ~ | ~ | ~ | ~ |

*Tests performed on binary cuts of ordinal outcomes. †Includes a composite of stable and unstable angina as a level. ‡Includes stable and unstable angina as separate levels.

Adj.: adjusted; Bin Chi: Binary Chi-square test; BLR: binary logistic regression; BS: bootstrapping; CAT: Cochran-Armitage trend test; CPH: Cox proportional hazards; Fatal Chi: Chi-square test performed on binary Fatal event/no event outcome; MACE: Major Adverse Cardiovascular event; Median: median test; MI: myocardial infarction; MLR: multiple linear regression; MWU: Mann-Whitney U test; OLR: ordinal logistic regression; Ord. Chi: Ordinal Chi-square test; SAE: Serious Adverse Event; VTE: Venous thromboembolism; WR: win ratio test.

**Key:** p >0.1 (~), 0.05-0.09 (+), 0.01-0.049 (++), 0.001-0.0099 (+++), <0.001 (++++).

Table 4. P-values of tests for each outcome in Minor stroke/ TIA participants recruited within 24 hours.

| Outcome | N | Levels | MWU | OLR | Median | WR | Adj. OLR | Ord. Chi | CAT | t-test | BS | Adj. MLR | Fatal Chi* | Adj. CPH* | CPH* | Adj. BLR* | Bin Chi* |
| --- | --- | --- | --- | --- | --- | --- | --- | --- | --- | --- | --- | --- | --- | --- | --- | --- | --- |
| Stroke, including TIA | 755 | 4 | ++ | ++ | ++ | ++ | ++ | + | ++ | ++ | ++ | + | ~ | ~ | ~ | ~ | ~ |
|  |  | 5 | ++ | ++ | ++ | ++ | ++ | + | ++ | ++ | ++ | + | ~ | ~ | ~ | ~ | ~ |
| (Primary outcome) |  | 6 | ++ | ++ | ++ | ++ | ++ | ~ | ++ | ++ | ++ | + | ~ | ~ | ~ | ~ | ~ |
|  |  | 9 | ++ | ++ | ++ | ++ | ++ | ~ | ++ | ++ | ++ | + | ~ | ~ | ~ | ~ | ~ |
| Stroke | 755 | 3 | ~ | ~ | ~ | ~ | ~ | ~ | ~ | ~ | ~ | ~ | ~ | ~ | ~ | ~ | ~ |
|  |  | 4 | ~ | ~ | ~ | ~ | ~ | ~ | ~ | ~ | ~ | ~ | ~ | ~ | ~ | ~ | ~ |
|  |  | 5 | ~ | ~ | ~ | ~ | ~ | ~ | ~ | ~ | ~ | ~ | ~ | ~ | ~ | ~ | ~ |
|  |  | 8 | ~ | ~ | ~ | ~ | ~ | ~ | + | + | ~ | ~ | ~ | ~ | ~ | ~ | ~ |
| MI | 755 | 3 | ~ | ~ | ~ | ~ | ~ | ~ | ~ | ~ | ~ | ~ | ~ | ~ | ~ | ~ | ~ |
|  |  | 4 | ~ | ~ | ~ | ~ | ~ | ~ | ~ | ~ | ~ | ~ | ~ | ~ | ~ | ~ | ~ |
|  |  | 5 | ~ | ~ | ~ | ~ | ~ | ~ | ~ | ~ | ~ | ~ | ~ | ~ | ~ | ~ | ~ |
| MI, including angina | 755 | 4† | ~ | ~ | ~ | ~ | ~ | ~ | ~ | ~ | ~ | ~ | ~ | ~ | ~ | ~ | ~ |
|  |  | 5† | ~ | ~ | ~ | ~ | ~ | ~ | ~ | ~ | ~ | ~ | ~ | ~ | ~ | ~ | ~ |
|  |  | 5‡ | ~ | ~ | ~ | ~ | ~ | ~ | ~ | ~ | ~ | ~ | ~ | ~ | ~ | ~ | ~ |
|  |  | 6† | ~ | ~ | ~ | ~ | ~ | ~ | ~ | ~ | ~ | ~ | ~ | ~ | ~ | ~ | ~ |
|  |  | 6‡ | ~ | ~ | ~ | ~ | ~ | ~ | ~ | ~ | ~ | ~ | ~ | ~ | ~ | ~ | ~ |
|  |  | 7‡ | ~ | ~ | ~ | ~ | ~ | ~ | ~ | ~ | ~ | ~ | ~ | ~ | ~ | ~ | ~ |
| Bleeding event | 756 | 3 | ++ | ++ | ++ | ++ | +++ | ++ | ++ | ++ | ++ | +++ | ~ | +++ | ++ | +++ | ++ |
|  |  | 4 | ++ | ++ | ++ | ++ | +++ | ~ | ++ | ++ | ++ | +++ | ~ | +++ | ++ | +++ | ++ |
|  |  | 5 | ++ | ++ | ++ | ++ | +++ | ~ | ++ | ++ | ++ | ++ | ~ | +++ | ++ | +++ | ++ |
| Cardiac event | 755 | 3 | ~ | ~ | ~ | ~ | ~ | ~ | ~ | ~ | ~ | ~ | ~ | ~ | ~ | ~ | ~ |
|  |  | 4 | ~ | ~ | ~ | ~ | ~ | ~ | ~ | ~ | ~ | ~ | ~ | ~ | ~ | ~ | ~ |
|  |  | 5 | ~ | ~ | ~ | ~ | ~ | ~ | ~ | ~ | ~ | ~ | ~ | ~ | ~ | ~ | ~ |
| SAE | 756 | 3 | ~ | ~ | ~ | ~ | ~ | ~ | ~ | ~ | ~ | ~ | ~ | ~ | ~ | ~ | ~ |
|  |  | 4 | ~ | ~ | ~ | ~ | ~ | ~ | ~ | ~ | ~ | ~ | ~ | ~ | ~ | ~ | ~ |
|  |  | 5 | ~ | ~ | ~ | ~ | ~ | ~ | ~ | ~ | ~ | ~ | ~ | ~ | ~ | ~ | ~ |
| MACE | 755 | 3 | ++ | ++ | ++ | + | ++ | + | ++ | ++ | ++ | ++ | ~ | ++ | ++ | ++ | ++ |
|  |  | 4 | ++ | ++ | ++ | + | ++ | ~ | + | + | + | + | ~ | ++ | ++ | ++ | ++ |
|  |  | 5 | ++ | ++ | ++++ | + | ++ | + | ++ | ++ | ++ | + | ~ | ++ | ++ | ++ | ++ |

*Tests performed on binary cuts of ordinal outcomes. †Includes a composite of stable and unstable angina as a level. ‡Includes stable and unstable angina as separate levels.

Adj.: adjusted; Bin Chi: Binary Chi-square test; BLR: binary logistic regression; BS: bootstrapping; CAT: Cochran-Armitage trend test; CPH: Cox proportional hazards; Fatal Chi: Chi-square test performed on binary Fatal event/no event outcome; MACE: Major Adverse Cardiovascular event; Median: median test; MI: myocardial infarction; MLR: multiple linear regression; MWU: Mann-Whitney U test; OLR: ordinal logistic regression; Ord. Chi: Ordinal Chi-square test; SAE: Serious Adverse Event; WR: win ratio test.

**Key:** p >0.1 (~), 0.05-0.09 (+), 0.01-0.049 (++), 0.001-0.0099 (+++), <0.001 (++++).
